# Evaluating the Efficacy of a Digital Therapeutic (CT-152) as an Adjunct to Antidepressant Treatment in Adults With Major Depressive Disorder: Protocol for the MIRAI Remote Study

**DOI:** 10.2196/56960

**Published:** 2024-08-20

**Authors:** Brian Rothman, Mary Slomkowski, Austin Speier, A John Rush, Madhukar H Trivedi, Erica Lawson, Michael Fahmy, Daniel Carpenter, Dalei Chen, Ainslie Forbes

**Affiliations:** 1 Otsuka Pharmaceutical Development & Commercialization, Inc Princeton, NJ United States; 2 Click Therapeutics, Inc New York, NY United States; 3 School of Medicine Duke University Durham, NC United States; 4 Duke-National University of Singapore Medical School Singapore Singapore; 5 Department of Psychiatry University of Texas Southwestern Medical Center Dallas, TX United States; 6 O’Donnell Brain Institute University of Texas Southwestern Medical Center Dallas, TX United States; 7 Otsuka Precision Health, Inc Princeton, NJ United States

**Keywords:** adherence, digital placebo, sham control, cognitive-emotional training, Emotional Faces Memory Task, mobile phone

## Abstract

**Background:**

Major depressive disorder (MDD) is common worldwide and can be highly disabling. People with MDD face many barriers to treatment and may not experience full symptom relief even when treated. Therefore, new treatment modalities are needed for MDD. Digital therapeutics (DTx) may provide people with MDD an additional treatment option.

**Objective:**

This study aimed to describe a phase 3 remote, multicenter, randomized, masked, sham-controlled trial evaluating the efficacy of a smartphone app–based DTx (CT-152) in adult participants diagnosed with MDD, used as an adjunct to antidepressant therapy (ADT).

**Methods:**

Participants aged 22-64 years with a current primary diagnosis of MDD and an inadequate response to ADT were included. Participants were randomized 1:1 to CT-152 or a sham DTx. CT-152 is a smartphone app–based DTx that delivers a cognitive-emotional and behavioral therapeutic intervention. The core components of CT-152 are the Emotional Faces Memory Task exercises, brief lessons to learn and apply key therapeutic skills, and SMS text messaging to reinforce lessons and encourage engagement with the app. The sham DTx is a digital working memory exercise with emotionally neutral stimuli designed to match CT-152 for time and attention. Participants took part in the trial for up to 13 weeks. The trial included a screening period of up to 3 weeks, a treatment period of 6 weeks, and an extension period of 4 weeks to assess the durability of the effect. Sites and participants had the option of an in-person or remote screening visit; the remaining trial visits were remote. Efficacy was evaluated using the Montgomery-Åsberg Depression Rating Scale, the Generalized Anxiety Disorder-7, Clinical Global Impression–Severity scale, the Patient Health Questionnaire-9, and the World Health Organization Disability Assessment Schedule 2.0. The durability of the effect was evaluated with the Montgomery-Åsberg Depression Rating Scale and Generalized Anxiety Disorder-7 scale. Adverse events were also assessed. Satisfaction, measured by the Participant and Healthcare Professional Satisfaction Scales, and health status, measured by the EQ-5D-5L, were summarized using descriptive statistics.

**Results:**

This study was initiated in February 2021 and had a primary completion date in October 2022.

**Conclusions:**

This represents the methodological design for the first evaluation of CT-152 as an adjunct to ADT. This study protocol is methodologically robust and incorporates many aspects of conventional pivotal pharmaceutical phase 3 trial design, such as randomization and safety end points. Novel considerations included the use of a sham comparator, masking considerations for visible app content, and outcome measures relevant to DTx. The rigor of this methodology will provide a more comprehensive understanding of the effectiveness of CT-152.

**Trial Registration:**

ClinicalTrials.gov NCT04770285; https://clinicaltrials.gov/study/NCT04770285

**International Registered Report Identifier (IRRID):**

RR1-10.2196/56960

## Introduction

Depression is a disabling and common disorder that affected 279.6 million people (5% of adults) worldwide in 2019 [1-3]. Major depressive disorder (MDD) accounted for 32.8 million years lived with disability globally in 2017, a 12.6% increase from 2007, and is predicted to be the leading global cause of disability by 2030 [4,5].

Early effective treatment of MDD is associated with better outcomes, and decreasing the duration of the depressive episode can reduce symptoms, improve clinical and functional outcomes, and increase the chance of full symptom relief [6-8]. However, people with MDD face many barriers to antidepressant therapy (ADT) and psychotherapy treatment, including access to mental health care professionals (HCPs) [9,10], logistical challenges such as long wait times for appointment scheduling and limited schedules due to work and family demands [11], and the stigma associated with mental illness [12]. In 2022, only 61.5% of US adults with a major depressive episode (MDE) in the past year received treatment for depression [13]. Global treatment rates are lower, with only 34.8% of people with depression receiving treatment annually [14].

Even when people with MDD can access treatment, it is not always successful. Many people with MDD do not respond to or achieve remission with ADT alone [15]. In the foundational Sequenced Treatment Alternatives to Relieve Depression (STAR*D) trial, only 48.6% and 36.8% of participants showed treatment response and achieved remission after first-line ADT, respectively, with lower percentages responding and remitting at each subsequent treatment step [15]. Psychotherapy may also not be effective for all patients; a meta-analysis of 193 studies of psychotherapy for the treatment of depression found a response rate of 41% at a 2-month follow-up [16]. Although ADT and psychotherapy alone may not work for all patients, data support that combining the 2 approaches is more effective for treating MDD symptoms than either approach alone [17-19].

Current treatment options may be difficult to access and are not effective for all people with MDD; therefore, new approaches for treating MDD are needed. One approach that may address this unmet need is the use of digital therapeutics (DTx), a subset of digital health [20-24]. DTx are software programs that deliver evidence-based interventions for medical conditions [25]. DTx may address a medical condition, manage, prevent, or treat a medical disorder or disease, or optimize medication [25]. When used to treat mental disorders, DTx may help overcome some barriers to treatment [26]. These software programs can be accessed remotely, allowing people with MDD more options than what may be available locally, and to access treatment on their own schedule [27-29]. Remote access to treatment can also ease stigma concerns and allow for more privacy [30]. DTx may offer an additional option for nonpharmacological treatment, expanding patient choice [31]. However, there are different types of digital health products in the United States that require different levels of regulatory oversight. Products that claim a treatment benefit must be cleared by the US Food and Drug Administration (FDA) and may require a prescription [25,32,33].

Otsuka Pharmaceutical Development & Commercialization, Inc, in collaboration with Click Therapeutics, Inc, has developed a smartphone app–based DTx, CT-152, for depression, used as an adjunct to ADT with the goal of reducing symptoms of depression. The core components of CT-152 are evidence-based training exercises (Emotional Faces Memory Task [EFMT]), brief lessons and activities to learn and apply key therapeutic skills, and supportive SMS text messaging to reinforce lessons and encourage engagement with the app. Although there are no comparable FDA-authorized digital therapeutics for MDD in the United States, there are some digital tools designed to support patients with MDD in addition to standard treatment that are available under FDA enforcement discretion (software that meets the regulatory definition of a device but pose minimal risk to patients, which can be obtained without a prescription). These tools have psychotherapy-based components but do not have training exercises [34]. The EFMT component of CT-152 was developed to adjust the connectivity in the affective network and regions of the cognitive control network that provide cognitive control over emotions [35,36].

A growing body of research has linked MDD with impaired cognitive control of emotionally salient information [37,38]. Impaired cognitive control is believed to manifest in perseverative thinking patterns (eg, rumination) [39]; these patterns serve to maintain or exacerbate the sad or depressed mood component in MDD [37-39]. Cognitive-emotional training, such as EFMT, has been shown to enhance cognitive control over emotions in people with MDD [40].

The goal of the EFMT exercises is to modify connectivity between the amygdala and dorsolateral prefrontal cortex by simultaneous emotion recognition and working memory tasks [36,37]. In 2 randomized controlled trials (RCTs), an improvement in depression symptoms was found to be statistically significant for participants with MDD who completed a digital EFMT regimen (4 and 6 weeks, respectively) compared with control participants who were not being treated with ADT or cognitive behavioral therapy (CBT) [36]. A follow-up study with participants who completed the 6-week EFMT regimen indicated that EFMT use altered connectivity between the amygdala with the right dorsolateral prefrontal cortex and bilateral dorsal anterior cingulate cortex; these altered connections correlated with improved symptoms [40].

The MIRAI trial (ClinicalTrials.gov NCT04770285) was designed to measure the effectiveness and safety of CT-152 for reducing depressive symptoms in patients diagnosed with MDD who were on ADT monotherapy for the treatment of depression. Data from MIRAI supported the FDA application for this product to be cleared as a prescription DTx for the treatment of people with MDD. There is also the potential to use these data to support regulatory approvals in other global markets.

MIRAI had design components reflecting conventional phase 3 pharmaceutical trials and novel components designed for DTx evaluation specifically. Conventional components included a control group and the evaluation of both effectiveness and safety. Effectiveness end points were established from gold-standard scales that are acceptable to the FDA [41-43]. Safety was evaluated by the frequency and severity of adverse events (AEs). Novel considerations in MIRAI included a sham comparator. The sham DTx consisted of a digital working memory exercise with emotionally neutral stimuli and was matched to CT-152 for time, attention, and expectation of benefit. The sham DTx also had an SMS text message component. Another consideration was treatment masking, which is important when designing studies for DTx [44]. Both participants and sites were masked to the treatment assignment. In DTx trials, a participant can spontaneously speak to an investigator, clinical raters, or study site staff about the exercises on the app (eg, participants reporting having difficulty with recognizing emotions on faces), leading to unmasking treatment assignment and introducing bias. Thus, additional masking considerations are required for both participants and investigators; steps taken in the MIRAI trial are listed in the Methods section. Finally, MIRAI incorporated outcomes that are relevant to DTx, including participant satisfaction with the treatment and adherence metrics.

There is a growing body of literature calling for DTx to be held to the same evidentiary standards as pharmacotherapy [45-50]. It is critical that the effectiveness and safety of these novel DTx are evaluated in RCTs with adequate sample sizes and clinically meaningful end points [51-53]. This trial will extend the findings from earlier EFMT studies and provide data on the efficacy and safety of this DTx. If shown to be effective, CT-152 may be considered a novel nonpharmacological option for people diagnosed with MDD to use as an adjunct to ADT. Here, we review the rationale and study design for the MIRAI trial, including procedures to enable a methodologically rigorous investigation of the new treatment options offered with DTx.

## Methods

### Overview

**Figure 1:**
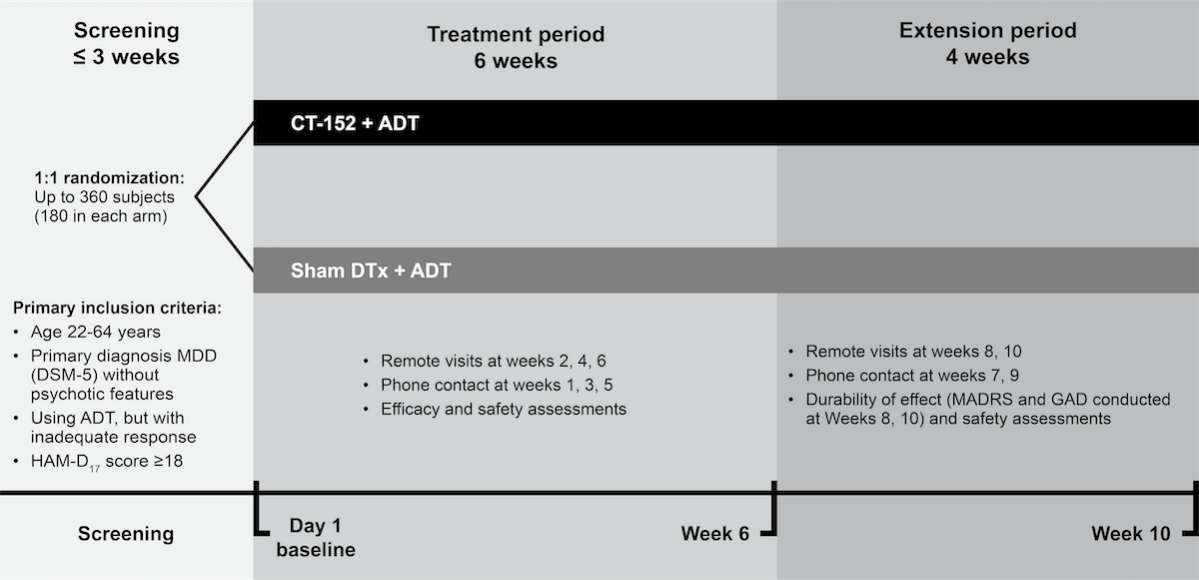
MIRAI Trial design.

This was a phase 3 multicenter, randomized, masked, sham-controlled trial to evaluate the efficacy of CT-152 in adult participants diagnosed with MDD who were taking ADT. Participants took part in the trial for up to 13 weeks. The trial included a screening period of up to 3 weeks, a treatment period of 6 weeks, and an extension period of 4 weeks to assess the durability of the effect (Figure 1). Given the COVID-19 pandemic and the advantages of remote trials, especially for a DTx, MIRAI was run as a remote study. Except the screening visit, all visits took place remotely; the screening visit had an in-person or remote option per investigator and patient preference. Due to the DTx nature of this trial, participants needed routine access to their smartphones in their work and home life.

### Study Participants

Key participant inclusion and exclusion criteria are listed in Textbox 1.

**Key inclusion and exclusion criteria for participation.**

**Inclusion criteria**

- Aged 22 to 64 years at the time of informed consent.
- Fluent in written and spoken English.
- Taking antidepressant therapy (ADT) monotherapy with a current primary diagnosis of major depressive disorder (MDD) based on the criteria in the DSM-5 (Diagnostic and Statistical Manual of Mental Disorders [Fifth Edition]), single or recurrent episode, without psychotic features, and did not meet the criteria for MDD with mixed features subtype.
- Had a score for the Hamilton Rating Scale for Depression, 17-item of ≥18 at screening and the baseline visit (day 1).
- Had a reported history of inadequate response to an adequate trial of ADT in the current episode.
  Inadequate response was defined as a reduction in depression symptom severity <50% per the Massachusetts General Hospital-Antidepressant Treatment Response Questionnaire (MGH-ATRQ).
  An adequate trial was defined as at least 6 weeks at a minimum therapeutic dose (or higher), according to the MGH-ATRQ.
- Were willing to maintain their current ADT at the current dose for the duration of their participation in the trial.
- Were currently using a smartphone not shared with anyone else, with an Android operating system 9.0 or greater capabilities or with iPhone Operating System 13.0 (Apple, Inc) or greater capabilities and agreed to download and use the smartphone app as required by the protocol.
- Had completed the onboarding module in the smartphone app during the screening period, continued consent to participate in the trial, and understood how to use the app at the baseline visit.

**Exclusion criteria**

- Had a reported inadequate response to >1 adequate trial of ADT (as defined in the inclusion criteria) for the current major depressive episode (MDE).
- Had been treated with psychopharmacological augmentation (eg, lithium, triiodothyronine, antipsychotics added to ADT, and multiple ADT) for any past episode or the current episode.
- Had received psychotherapy within 90 days before screening.
- Had significant suicidal ideation within the last year, suicidal behavior within the past 2 years, or presented a serious risk of suicide (in the opinion of the investigator).
- Had been treated with electroconvulsive therapy or neuromodulation devices (ie, transcranial magnetic stimulation, vagus nerve stimulation, or transcranial direct current stimulation) for MDD in the past.
- Were currently using a computer, web, or smartphone software–based application or equivalent for mental health or MDD; participants could enroll if this app was discontinued before the baseline visit.
- Had been in a current MDE lasting longer than 2 years.
- Were considered resistant or refractory to treatment by history and per investigator judgment.
- Had a lifetime diagnosis of schizophrenia, schizoaffective disorder, other psychotic disorder, bipolar I or II disorder, or current posttraumatic stress disorder, panic disorder, or obsessive-compulsive disorder.
- Had been diagnosed with any DSM-5 personality disorder.
- Had MDD due to a general medical condition or a neurologic disorder.

### Ethical Considerations

This trial was conducted in accordance with local laws, the International Conference on Harmonization Good Clinical Practice guidelines, and the Declaration of Helsinki. The protocol was reviewed and approved by the governing institutional review board (IRB) for each investigational site. Of the 54 study sites, IRB services were provided by Advarra (approval number 00000971) for 53 sites, and WCG IRB (approval number 1-1377994-1) for 1 site. IRB approvals were granted between November 2020 and January 2021. All participants were asked to provide consent to participate in this study before enrollment and agree to its privacy policy and terms of service required for the remote collection of their information. All information collected in the trial was considered confidential and was only used in accordance with the protocol. Participants were identified only by a unique identification number in eSource. However, regulatory officials or sponsor personnel could be allowed access to records, consistent with local requirements. Participants could withdraw from the trial at any time without justification. Participants received a stipend to help cover the costs associated with being in the study. Participants also had the option to have their ADT provided to them by the sponsor at no charge for the length of the study.

### Intervention

CT-152 is a smartphone app–based DTx that delivers a proprietary interactive cognitive-emotional and behavioral intervention, intended for use as an adjunct to ADT. The core components of CT-152 are the EFMT exercises; brief CBT-based lessons, each paired with an out-of-app activity or guided audio psychotherapy exercise targeting the most common MDD symptoms; and SMS text messaging to reinforce the CBT-based lessons and encourage engagement with the app. Each treatment session consisted of an EFMT exercise and a brief lesson with an activity. During the EFMT exercises, participants were presented with a series of pictures of faces, tasked with identifying the emotions they observed and remembering the sequence of emotions [35]. Using an N-back working memory training paradigm, for each face observed, participants indicated whether the emotion is the same as the emotion N faces back [35]. To date, CT-152 has only been developed in English.

A sham DTx served as a control and provided a cognitive training exercise designed to retain user interest while minimizing therapeutic effects. Each sham session consisted of a Shapes Memory Task (SMT) exercise. The SMT is a working memory task that provides users with an analogous structure, matched for time and attention, to the cognitive-emotional training exercise (EFMT) in CT-152. The sham DTx did not include EFMT exercises or lessons to retain its intended nontherapeutic nature. Participants in the control group also received supportive SMS text messages related to maintaining engagement with study treatment. The SMT exercise has been previously used in EFMT pilot studies as the control condition [35,36].

In order to be eligible to participate, during the screening period, participants had to complete an N-back working memory task simulating basic features of CT-152 to ensure they had the necessary skills to use it. This also confirmed their interest in participating in the study. After eligibility was confirmed, participants were randomized 1:1 to either CT-152 or sham DTx at the baseline visit (day 1). The participants progressed through a treatment schedule of 18 sessions at a rate of 3 sessions per week over the 6-week treatment period (day 1 to week 6). Participants had a remote study visit at weeks 2, 4, and 6 and were contacted by telephone at weeks 1, 3, and 5.

After week 6, participants continued in the trial during the extension period (weeks 7 to 10) to assess the durability of the effect. In the extension period, the smartphone app remained installed for each group; no new therapeutic content was introduced, and EFMT and SMT exercises were not available. The 2 groups each received brief supportive SMS text messages in the extension period with content related to the previously completed exercises. Participants had a remote study visit at weeks 8 and 10 and were contacted by the trial site by telephone at weeks 7 and 9.

A dedicated call center was available to support the participants and the trial sites on the initial downloading of and access to the smartphone app, as well as any technical issues with the app throughout the trial.

### Outcomes and Assessments

The primary objective of the trial was to evaluate the efficacy of CT-152 in improving depressive symptoms versus the sham DTx, which served as a control. The primary efficacy end point was the change from baseline to week 6 in the Montgomery-Åsberg Depression Rating Scale (MADRS) total score.

Other efficacy end points included assessment of the MADRS at weeks 2 and 4; Generalized Anxiety Disorder-7 (GAD-7) scores at weeks 2, 4, and 6; the Clinical Global Impression–Severity scale scores (CGI-S) at weeks 2, 4, and 6; and the Patient Health Questionnaire-9 (PHQ-9) scores at weeks 4 and 6. Functional outcomes were assessed by World Health Organization Disability Assessment Schedule 2.0 (WHODAS 2.0) scores and quality of life by EQ-5D-5L scores at week 6. To evaluate the durability of the effect of the DTx, the change from baseline in MADRS and GAD-7 total score as well as MADRS response and remission rates were evaluated at weeks 8 and 10. The MADRS was conducted by trained and calibrated independent raters. Trial site staff administered the CGI-S. Participants completed the GAD-7, PHQ-9, WHODAS 2.0, and EQ-5D-5L (for scale descriptions, refer to Table 1).

**Table 1:** Scales used for study assessments.

Scales	Description
MGH-ATRQ^a^	A 6-item, participant self-reported scale to evaluate treatment-resistant depression [54,55]
HAM-D_17_^b^	A 17-item, observer-reported scale to evaluate depression severity and response to treatment [56]
MADRS^c^	A 10-item, HCP^d^-reported scale to evaluate depression severity [57]
CGI-S^e^	A single-item, observer-reported scale to evaluate the severity of mental illness over the past week [58]
PHQ-9^f^	A 9-item, participant self-reported scale to screen for and assess depression severity [56]
GAD-7^g^	A 7-item, participant self-reported scale to screen for and assess the severity of general anxiety disorder [59]
WHODAS 2.0^h^	A 36-item, participant self-reported scale to assess the impact of a health condition on functioning [60]
Participant satisfaction scale	A 9-item, participant self-reported scale designed for the MIRAI trial to assess participants’ satisfaction with their DTx^i^ assignment for the trial
HCP satisfaction scale	A 2-item, HCP self-reported scale designed for the MIRAI trial to assess HCP satisfaction with delivering the DTx used by their patients during the trial
EQ-5D-5L	A 5-item, participant self-reported scale to measure overall health status. It includes a vertical visual analog scale for participants to indicate their perception of their overall health [61]
C-SSRS^j^	A 6-item, participant self-reported scale to assess suicide risk, severity of risk, and support needed [62]

^a^MGH-ATRQ: Massachusetts General Hospital-Antidepressant Treatment Response Questionnaire.

^b^HAM-D_17_: Hamilton Rating Scale for Depression, 17-item.

^c^MADRS: Montgomery-Åsberg Depression Rating Scale.

^d^HCP: health care professional.

^e^CGI-S: Clinical Global Impressions-Severity.

^f^PHQ-9: Patient Health Questionnaire-9.

^g^GAD-7: Generalized Anxiety Disorder-7.

^h^WHODAS: World Health Organization Disability Assessment Schedule.

^i^DTx: digital therapeutics.

^j^C-SSRS: Columbia-Suicide Severity Rating Scale.

The safety objective was to assess the safety of CT-152 in adult participants with MDD who were concurrently taking ADT. Safety was evaluated by the frequency and severity of AEs, serious AEs, and discontinuations from the trial due to AEs. AEs related to smartphone use may include dizziness, fatigue, or headache and were anticipated as potential AEs reported by the participants [63,64]. Previous research did not assess AEs related to EFMT use [35,36]. Participants were required to be on ADT monotherapy, and the safety profile of ADTs used for MDD is well understood. Based on the known risks of ADT and smartphone use and a preliminary understanding of the risks of EFMT use, no additional risks were anticipated as related to CT-152 use. All participants were assessed weekly for both anticipated and unanticipated AEs; any participants reporting serious AEs were monitored.

Participant adherence was monitored weekly. Furthermore, the number of CT-152 or sham sessions completed over the entire trial course was recorded.

### Masking Considerations

In addition to masking the participants and trial sites for the treatment assignment, masked independent raters conducted the MADRS assessment by telephone. The independent raters had no access to trial procedures, treatment assignment, trial data, or clinical information other than what was solicited for the assessment. In addition, both independent raters and participants were instructed to avoid discussion of the DTx content and other aspects of the trial.

To minimize potential bias, trial sites had designated administrators of the other efficacy assessments. Administrators had limited contact with the participants and did not perform adherence checks. Participants completed self-reported outcomes independently and remotely.

The hypothesis of this trial—CT-152 would lead to greater improvement in MDD symptoms versus the sham DTx—was masked, and participants were informed that either DTx may be effective. This approach was intended to optimize adherence in both arms and limit the risk of participant unmasking and associated expectation biases. At the end of the study, participants were informed of the trial hypothesis, but were not informed of which DTx they had received.

CT-152 meets the definition of a nonsignificant risk device per 21 Code of Federal Regulations § 812.3(m) and a low- to moderate-risk device per Software as a Medical Device [65,66]. The masking approaches used in this trial were only used because (1) CT-152 posed minimal risks, (2) masking did not adversely affect the participants, and (3) the trial could only be conducted by withholding the information on participant treatment assignment.

### Sample Size Calculation

The initial sample size was calculated to detect a 3-unit difference between the CT-152 plus ADT and sham DTx plus ADT in the change from baseline in MADRS total score, with 85% power at a 2-sided α=.05 level. The resulting sample size was 324 evaluable participants in total (162 in each arm). The full analysis set was defined as all randomized participants who received at least 1 CT-152 or sham DTx session and had at least 2 MADRS total score assessments, 1 of which was at baseline. To compensate for participants who failed to have evaluable assessments of MADRS total score in the full analysis set sample (estimated at up to 10% of all participants), a total of approximately 360 participants (180 in each arm) were planned to be randomized. The trial randomized eligible participants 1:1 across 54 sites. The sample size at any single trial site was capped at approximately 15% of the total number of randomized participants. Randomization was stratified by the trial site.

Due to the limitations of applying assumptions on the treatment effect size, and to ensure adequate power of the trial, an unmasked interim analysis was conducted by a data monitoring committee, whose members recommended continuing the trial with the planned sample size of 360 participants (actual enrollment included 386 participants).

### Statistical Methods

Baseline and demographic characteristics, including age, sex, race, and ethnicity, were summarized using descriptive statistics.

#### End Points and Assessments

The primary efficacy analysis was based on the mixed-model repeated measurements method and the change from baseline score to week 6 for the MADRS. The MADRS score change from baseline to weeks 2 and 4 was also assessed. Additional efficacy analyses included changes from baseline to predetermined end points for the GAD-7, CGI-S, WHODAS 2.0, and PHQ-9. MADRS partial and full response rates (≥30% to <50% from baseline and ≥50% reduction from baseline, respectively) at weeks 2, 4, and 6 were analyzed; MADRS response rates were also analyzed during weeks 8 and 10 of the extension period. All efficacy end points were based on the full analysis set.

The durability of the treatment effect was assessed based on MADRS and GAD-7 scores during the extension period. The SAS Enterprise Guide version 8.3 (SAS Institute Inc.) was used for statistical analyses.

#### Plan for Missing Data in Efficacy End Points

All data collected during the trial treatment period and extension period were used for statistical analysis. The mixed-model repeated measurements method assumed data were missing at random, which is a reasonable assumption for longitudinal clinical trials in MDD [67]. However, the possibility of missing not at random data was also considered. As sensitivity analyses, pattern-mixture models were used to explore data missing mechanisms of missing not at random and investigate the response profile of participants by dropout reason.

#### Exploratory End Points

MADRS remission rates at weeks 2, 4, and 6 were analyzed. Remission was defined as a MADRS total score of ≤10 and a decrease of 50% or more of the total score from baseline. Satisfaction, measured by the Participant and Healthcare Professional Satisfaction Scales, and health status, measured by the EQ-5D-5L, were summarized using descriptive statistics.

#### Safety End Points

All AEs were coded by system organ class and Medical Dictionary for Regulatory Activities (MedDRA)-preferred term. The incidence of the following events was summarized by treatment group: (1) AEs that are potentially causally related to the treatment or sham DTx, (2) AEs with an outcome of death, serious AEs, and (3) AEs leading to discontinuation from the trial.

#### Adherence

Adherence was evaluated for all participants as the percentage of sessions completed over the course of the trial.

## Results

The MIRAI study was funded in September 2020. It was initiated in February 2021, with a primary completion date of October 2022. As of study completion, 386 participants were enrolled. The primary data analysis is complete and expected to be published in the fall of 2024.

## Discussion

### Principal Findings

This was the first investigation of CT-152 as an adjunct to ADT for the treatment of depression. CT-152 has both cognitive-emotional training and behavioral intervention components, which is a novel combination compared with other digital tools designed to support patients with MDD currently available under FDA enforcement discretion [34]. This protocol incorporated aspects of conventional phase 3 pharmaceutical trials, including using a randomized controlled design and gold-standard scales for evaluating effectiveness. In addition, the safety analyses were intended to evaluate if CT-152, designed to provide a clinical intervention, had a low safety risk and was well tolerated by participants. MIRAI also considered factors that are not typical for DTx study design. The use of a sham DTx comparator minimized the potential that any changes between groups were because of CT-152 and not due to the demand characteristics of using a DTx. Bias related to knowledge of study treatment assignment was managed through robust masking procedures. Adherence was evaluated for both CT-152 and the sham DTx arms.

### Safety

Although DTx for mental disorders may be less likely to lead to AEs than pharmacotherapy, AEs, such as dizziness, fatigue, and headache, that are associated with mobile device use may also be associated with DTx [63,64,68]. Therefore, it is important to assess the safety of DTx. Other current clinical trials for DTx for MDD do not assess safety outcomes [69]. Among 11 protocols for clinical trials assessing DTx for MDD published recently, only 1 mentions assessing AEs along with suicide risk [70]; 5 others assess suicide risk only [71-75]. Unlike the pivotal trials for current FDA-cleared DTx for mental disorders, the MIRAI trial assessed safety outcomes [76-79]. CT-152 was expected to be well tolerated due to the digital delivery of therapy and the mechanisms of action of its core therapeutic modules.

### Sham Comparator

In trials for pharmacological treatments, masking is typically achieved through a placebo that resembles the treatment being tested. In RCTs for DTx, having a placebo that allows for masking can be challenging [50]. Of 11 clinical trial protocols published in the past few years that evaluated digital interventions for MDD, only 5 involved masking; 1 of the 5 trials masked both participants and interviewers [80]. When a participant is waitlisted or given treatment as usual during a trial of digital intervention, they may become aware of not receiving the treatment under study, which could impact their involvement and expectations [81]. In conclusion, there are masking advantages to using a sham comparator.

The variability in comparator arms in DTx trials can lead to challenges in understanding the effects of digital treatments as a class and relative to one another. In total, 3 meta-analyses of digital interventions for depression found lower effect sizes in studies of smartphone interventions that included comparators that controlled for time and attention but did not provide therapeutic intervention, compared with those studies with inactive comparators or waitlist controls [81-83]. This suggests that smartphone use alone may confer psychological benefits [46,81-84]. Using a digital comparator may raise the evidentiary standard for evaluating a digital treatment [83,84]. Given these recent findings and the potential confounding factors when using a nondigital comparator for the control group (eg, waitlist or treatment as usual), a digital comparator is important for a more unbiased and accurate understanding of the effectiveness of a DTx [81-83].

The MIRAI trial design is innovative because it included a sham DTx as the comparator, which helps to ensure that the comparison reflected a true change in symptoms (if any), rather than a nonspecific benefit related to digital interventions more broadly [46,81,82]. The sham DTx more easily allowed for masking, with less risk of participants and investigators becoming unmasked.

### Outcomes Important for DTx

An important consideration for any treatment is understanding the relationship between improvement in patient symptoms and increased patient adherence to the treatment; this is a novel consideration for digital interventions [85,86]. For MDD specifically, among 11 study protocols involving DTx for MDD, 9 protocols described adherence assessments [80]. While data on adherence were collected, none of the protocols defined adherence or how it would be evaluated.

A systematic literature review on adherence and engagement with digital interventions for MDD defined adherence as the percentage of sessions completed (the number of sessions completed divided by the total number of sessions) and found that only 38 out of 94 studies reviewed had this information available [80]. Their findings highlight the importance of establishing a standardized metric for adherence to digital interventions to better compare results across studies and different types of interventions [80]. The MIRAI study was designed to collect data that can be used to measure the percentage of sessions completed, which will contribute to the body of literature measuring adherence to digital interventions in a way that can be compared across studies.

Another novel consideration for DTx that does not often apply to pharmacological treatments is the consideration of patient satisfaction with and the ability to easily use the intervention. This can include features such as an easy onboarding practice, personalizing app content, and text reminders for patients [87]. CT-152 includes text messages to remind participants of what they learned and to engage them with treatment. In addition, MIRAI had both a participant and an HCP satisfaction survey to gain further insights into how easy CT-152 was to use and how satisfied participants were with their experience.

### Limitations

While this study design addressed many common concerns about trials for digital interventions, it had limitations, such as not including adults younger than 22 years or older than 65 years. Therefore, potential benefits for these populations will need to be explored in future research [88]. The inclusion criterion for MDD severity was a Hamilton Rating Scale for Depression, 17-item score of ≥18, potentially excluding participants with milder MDD. A further limitation is that the MIRAI trial assessed CT-152 as an adjunct to ADT, and thus the results cannot be generalized to using the digital intervention without ADT.

There were also potential limitations related to remote trials and digital interventions, such as limited access to technology or low digital literacy. In this study, participants were required to own a smartphone and had to complete an onboarding module to show they had an interest in study participation and the skills necessary to use CT-152 or the sham DTx. These requirements limited the potential for low digital access or literacy to impact participation.

In addition, a common occurrence in trials for digital interventions or that use remote data collection is participant attrition [89,90]. Although most clinical trials have some participants discontinue or be unresponsive to follow-up, this is more likely to happen in studies involving DTx [89,90]. Participant attrition was evaluated after the trial but was expected to be similar to what is seen in the literature. Participant engagement and adherence to digital interventions were also concerns [80]. Both CT-152 and the sham DTx had text messages designed to encourage participant adherence and engagement throughout the study. To encourage overall continued adherence to the study program, participants were informed before enrolling that remote visits would be required, and they reviewed the visit schedule with trial site staff once randomized to a treatment arm. Adherence checks were also performed during the treatment period and adherence was monitored remotely through the DTx. Any signs of nonadherence were addressed by the investigator to remind and encourage participants to continue adhering to the study program.

### Conclusions

DTx has the potential to address many barriers to MDD treatment, including increasing access to more treatment options, allowing for remote access, easily fitting into a patient’s schedule, and easing stigma concerns [26-28,30]. However, it is important that the effectiveness and safety of DTx are evaluated in RCTs with rigorous study design [51-53].

Many considerations in conventional phase 3 trial design, such as safety end points, adherence, and the use of a sham comparator, have been incorporated into this protocol to assess the efficacy of CT-152 for MDD, although the way these considerations are assessed is often different for pharmacological treatment. Incorporating safety and adherence considerations, along with participant masking to the treatment, are not yet typical for DTx evaluation. Moreover, rigorous assessments of these outcomes will provide a more comprehensive understanding of the effectiveness of CT-152.

## Abbreviations

ADT: antidepressant therapy
AE: adverse event
CBT: cognitive behavioral therapy
CGI-S: Clinical Global Impression–Severity scale
DTx: digital therapeutics
EFMT: Emotional Faces Memory Task
FDA: US Food and Drug Administration
GAD-7: General Anxiety Disorder-7 scale
HCP: health care professional
IRB: institutional review board
MADRS: Montgomery-Åsberg Depression Rating Scale
MDD: major depressive disorder
MDE: major depressive episode
MedDRA: Medical Dictionary for Regulatory Activities
PHQ-9: Patient Health Questionnaire-9
RCT: randomized controlled trial
SMT: Shapes Memory Task
STAR*D: Sequenced Treatment Alternatives to Relieve Depression
WHODAS 2.0: World Health Organization Disability Assessment Schedule 2.0
